# Pre-treatment Resting-State Functional MR Imaging Predicts the Long-Term Clinical Outcome After Short-Term Paroxtine Treatment in Post-traumatic Stress Disorder

**DOI:** 10.3389/fpsyt.2018.00532

**Published:** 2018-10-30

**Authors:** Minlan Yuan, Changjian Qiu, Yajing Meng, Zhengjia Ren, Cui Yuan, Yuchen Li, Meng Gao, Su Lui, Hongru Zhu, Qiyong Gong, Wei Zhang

**Affiliations:** ^1^Mental Health Center and Psychiatric Laboratory, West China Hospital of Sichuan University, Chengdu, China; ^2^Huaxi Brain Research Center, West China Hospital of Sichuan University, Chengdu, China; ^3^Department of Radiology, Huaxi MR Research Center, West China Hospital of Sichuan University, Chengdu, China; ^4^Radiology Department of the Second Affiliated Hospital, Wenzhou Medical University, Wenzhou, China

**Keywords:** posttraumatic stress disorder, pharmacotherapy, clinical outcome, amplitude of low-frequency fluctuations, degree centrality, support vector machine

## Abstract

**Background:** The chronic phase of post-traumatic stress disorder (PTSD) and the limited effectiveness of existing treatments creates the need for the development of potential biomarkers to predict response to antidepressant medication at an early stage. However, findings at present focus on acute therapeutic effect without following-up the long-term clinical outcome of PTSD. So far, studies predicting the long-term clinical outcome of short-term treatment based on both pre-treatment and post-treatment functional MRI in PTSD remains limited.

**Methods:** Twenty-two PTSD patients were scanned using resting-state functional MRI (rs-fMRI) before and after 12 weeks of treatment with paroxetine. Twenty patients were followed up using the same psychopathological assessments 2 years after they underwent the second MRI scan. Based on clinical outcome, the follow-up patients were divided into those with remitted PTSD or persistent PTSD. Amplitude of low-frequency fluctuations (ALFF) and degree centrality (DC) derived from pre-treatment and post-treatment rs-fMRI were used as classification features in a support vector machine (SVM) classifier.

**Results:** Prediction of long-term clinical outcome by combined ALFF and DC features derived from pre-treatment rs-fMRI yielded an accuracy rate of 72.5% (*p* < 0.005). The most informative voxels for outcome prediction were mainly located in the precuneus, superior temporal area, insula, dorsal medial prefrontal cortex, frontal orbital cortex, supplementary motor area, lingual gyrus, and cerebellum. Long-term outcome could not be successfully classified by post-treatment imaging features with accuracy rates <50%.

**Conclusions:** Combined information from ALFF and DC from rs-fMRI data before treatment could predict the long-term clinical outcome of PTSD, which is critical for defining potential biomarkers to customize PTSD treatment and improve the prognosis.

## Introduction

Posttraumatic stress disorder (PTSD) is a debilitating psychiatric disorder characterized by re-experiencing, avoidance, numbing, and hyperarousal (1), and is a major worldwide public health problem (2, 3). Antidepressants are the one of the predominant pharmacotherapies for PTSD. The selective serotonin reuptake inhibitors (SSRIs), sertraline and paroxetine, are approved by the Food and Drug Administration (FDA) for the treatment of PTSD based on multicenter trials (4, 5). However, studies were inconclusive regarding evidence of the efficacy of these drugs (6–8). The chronic phase of PTSD and the limited effectiveness of existing treatments combine to create an urgent need for the development of potential biomarkers to predict response to antidepressant medication at an early stage. Indeed, a number of potential biomarkers of neuroimaging have been proposed in recent years. For example, preliminary evidence showed that increased orbitofrontal cortex (OFC) function is specifically associated with paroxetine treatment in PTSD (9). More recently, we have demonstrated that regional spontaneous activity of the precuneus reflects the clinical improvement of patients, and manipulating the activity of the precuneus and OFC could be a prognostic indicator of PTSD (10). In addition, the least recruitment of prefrontal emotion regulatory brain regions has been associated with most reduction in PTSD symptoms with SSRI treatment (11).

These findings, however, investigate acute therapeutic effect and assess the symptom changes only immediately after treatments, without following up the long-term treatment outcome of PTSD. Moreover, none of these studies associated the post-treatment brain magnetic resonance imaging (MRI) features with treatment outcomes, although it was observed in previous single photon emission computed tomography study that the post-treatment activation in the medial prefrontal cortex region was correlated with Clinician-Administered PTSD Scale reduction (12). The post-treatment MRI contains brain functional changes after the shot-term treatment, and may as well provide useful information in predicting long-term treatment outcome. Many studies have emphasized the relapse prevention after acute treatment for PTSD (13, 14), therefore, it is important to be able to predict the long-term prognosis based on both pre-treatment and post-treatment MRI, guiding the clinicians and patients to make optimal treatment plans.

In the past several years, the application of machine learning techniques to neuroimaging data analysis has made promising improvements in brain disease classification (15, 16) or the prediction of remission in treated patients (17, 18). In contrast to the group comparisons that are based on mass-univariate analyses, machine learning techniques allow prediction of individual cases, and they are sensitive to subtle and spatially distributed differences in the brain (19). In clinical practice, machine learning techniques such as support vector machine (SVM) have considerable translational value. It has been demonstrated that when applying machine learning methods to characterize PTSD, the classification accuracy obtained using multi-level features from resting-state functional MRI (rs-fMRI) data was increased from the two-level and single-level feature–based methods, respectively (20). Rs-fMRI studies have identified that altered amplitude of low-frequency fluctuations (ALFF) (10, 21) and degree centrality (DC) (22), which represent regional spontaneous neural activity and the level of integration of that local activity across brain regions, respectively, underlie PTSD. Investigating the ALFF may advance our understanding of the functional segregation of the brain, while investigation on DC may increase our understanding of the functional integration within the brain (23). The combined levels of rs-fMRI data provide complementary information for classification, and may give better classification performance than single-level features (24).

Therefore, we used SVM to examine the long-term prognostic value of both pre-treatment and post-treatment rs-fMRI data in patients with PTSD. Specifically, ALFF and DC were used as classification features and effectively combined. We hypothesized that complementary information conveyed among regional and integrated features could be combined to discriminate between remitted patients and consistent patients in the long term with statistically significant accuracy. Since we found a significant correlation between the changes of mean ALFF and CAPS scores in the PTSD group before and after treatment in our previous study (10) and the 2-year follow-up data of the current study is continuation of our previous work (10), we also hypothesized that the post-treatment rs-fMRI would be more predictive than the pre-treatment rs-fMRI data.

## Materials and methods

### Participants

The sample of PTSD patients were a cohort of earthquake survivors, from our previous study, where detailed information of trauma events and inclusion/exclusion criteria were described (10). In brief, imaging data from 22 patients were included for statistical analysis (5 males and 17 females, with a mean age of 45.82 ± 7.01 years) before and after treatment. All individuals met the DSM-IV criteria for PTSD, right-handed. None of the patients had received any regular medication or psychological therapy before the first MRI scan. Any history of neurological disease or alcohol and/or other substance abuse/dependence; history of major head injury involving loss of consciousness for more than 10 min; pregnancy, serious systemic illness, MR imaging contraindications or mental retardation were excluded. Individuals with head motion of more than 1.5 mm or 1.5° during rs-fMRI were excluded. Diagnosis of PTSD was determined by consensus of two attending psychiatrists using the Clinician-Administered PTSD Scale (CAPS) (25) and the Structured Clinical Interview (SCID) Diagnostic and Statistical Manual of Mental Disorders, Fourth Edition (DSM-IV), Patients Version (26). All 22 participants were followed up using the SCID and CAPS 2 years after they underwent the second MRI scan, two of whom were unavailable for follow-up.

According to the SCID, participants met criteria for current comorbid diagnoses before treatment: major depression (*N* = 5), dysthymia (*N* = 1), and general anxiety disorder (*N* = 2); only two individuals were comorbid with depression during the follow-up period of 2 years.

### Treatment phase

As was described in our previous study (10), participants with PTSD received paroxetine (Seroxat) treatment for 12 weeks. The dosage at the beginning of treatment was 10 mg/day, which was increased to 20 mg/day after 4 days. The paroxetine dosage was adjusted based on the judgment of the investigating psychiatrist every 4 weeks by 10 mg/day up to 40 mg/day. The CAPS, Clinical Global Impression (CGI), Hamilton Rating Scale for Depression (HAMD-24), Hamilton Rating Scale for Anxiety (HAMA-14), and Asberg's antidepressant side-effect rating scales (SERS) were assessed every 4 weeks to evaluate the patient's condition and adverse drug reactions. Medication was dispensed during every visit to enhance compliance and reduce chance of misuse. The study psychiatrist and staff inquired about missed doses and conducted a pill count to confirm the participant's report. No participants in the study ever missed more than 2 consecutive daily doses and no subject regularly (>3 times) missed a dose over the course of the 12-week study. The 24/7 emergency telephone number of a psychiatrist was given to all subjects in case of any emergency during treatment. No other drugs were permitted during treatment, unless required for the patients' safety.

The 3-month pharmacotherapy was free of charge for all the participants, and after that, the participants chose to buy and continue taking paroxetine at their own expense. At follow-up, only two participants reported to have continued paroxetine for another 2 months. Other participants did not take any antipsychotic medication after the 3-month treatment for various reasons, primarily because of (1) living in remote mountain areas where antipsychotic medications were locally unavailable, (2) poor socioeconomic conditions that limited travel and funds for medical care, (3) feeling well after the 3-month pharmacotherapy and a lack of understanding or recognition of the severity of mental illness. No participants reported any other life events according to the Life Events Checklist during the follow-up period of 2 years. In addition, three patients reported use of complementary medical treatments with Chinese traditional medicine during the 2 years. Because the effects of the ingredients on brain structure and function were unknown and the three patients stopped taking the medicine for more than 6 months, we did not exclude them from the study.

This study was approved by the Medical Ethics Committee of West China Hospital, Sichuan University, and all subjects gave written informed consent.

### MR data acquisition and data preprocessing

All participants underwent rs-fMRI before and after pharmacotherapy with a 3.0-T MR imaging system (Siemens Trio Tim) and a twelve-channel phased-array head coil. Thirty transverse slices (field of view [FOV] = 24 cm, in-plane matrix = 64 × 64, slice thickness = 5 mm, no slice gap, voxel size = 3.75 × 3.75 × 5), aligned along the anterior commissure-posterior commissure (AC-PC) line, were acquired using an echo planar imaging (EPI) sequence (time repetition [TR] = 2,000 ms, time echo [TE] = 30 ms, flip angle = 90°), resulting in a total of 205 volumes for each participant. During imaging, the participants were fitted with soft ear plugs and instructed to relax with their eyes closed; without falling asleep; and without directed, systematic thought. Subsequently, high-resolution, three-dimensional T1-weighted images (TR = 1,900 ms, TE = 2.26 ms, flip angle = 9°, 176 sagittal slices with thickness = 1 mm, FOV = 240 × 240 mm^2^ and data matrix = 256 × 256, yielding an in-plane resolution of 0.94 × 0.94 mm^2^) were acquired.

Data processing was performed using the Data Processing Assistant for Resting-State fMRI (DPARSF) software package (http://rfmri.org/DPARSF) (27), implemented in MATLAB (MathWorks, Inc., USA). Considering the magnetization saturation effects and participants' adaptation to the environment, the first 5 volumes of each data set were discarded. Functional volumes were first slice-time corrected and then motion corrected. All participants in this study had <1.5 mm displacement and 1.5° of rotation in any direction. Moreover, examination of the movement parameters showed that there was no significant association between the mean displacement and long-term outcome (CAPS change) (pre-treatment *r* = 0.296, *p* = 0.181; post-treatment *r* = 0.063, *p* = 0.781) or initial CAPS (pre-treatment *r* = 0.031, *p* = 0.890; post-treatment *r* = −0.042, *p* = 0.852). The T1 images were registered to the averaged EPI image and then spatial normalization was performed to a 3-mm Montreal Neurological Institute template, and smoothed using a 6-mm, full-width half maximum (FWHM) Gaussian kernel (28, 29) for ALFF calculation. For DC calculation, smoothness was the last step after the DC was calculated to avoid disturbing correlations between these voxels, as the DC was based on Pearson's correlations between the time series of all pairs of brain voxels. The covariates of no interest, including white matter signal, cerebrospinal fluid signal, and the Friston 24-parameter model, were sequentially regressed from the time series (30). Global signal regression (GSR) was not performed because it might have removed neural (functionally relevant) BOLD signal and potentially altered group differences in functional connectivity (31, 32). The ALFF and the DC measures were calculated using DPARSF.

### ALFF calculation

After preprocessing, the corrected BOLD time series were transformed to the frequency domain using fast Fourier transform (FFT) (parameters: taper percent = 0; FFT length = shortest) to obtain the power spectrum. To calculate the ALFF, the power spectrum was square-rooted and averaged across 0.01–0.08 Hz at each voxel. Finally, the ALFF of each voxel was then divided by the global mean of ALFF values for standardization.

### Degree centrality calculation

After preprocessing, the corrected BOLD time series were low pass-filtered using a cut-off frequency of 0.08 Hz to reduce low frequency drift and high frequency. Furthermore, binarized DC measures were calculated using DPARSF. First, Pearson's correlations between the times series of all pairs of gray matter voxels were calculated to obtain a whole functional connectivity matrix for each participant. Second, we restricted our analysis to positive correlations above a threshold of Pearson's *r* = 0.25 to obtain an undirected binarized matrix according to previous studies (28, 33, 34). If the correlation between the two voxels was >0.25, the elements of the binarized matrix were set to 1; otherwise, they were set to 0 (33). This threshold avoids taking the voxels that had low temporal correlation into consideration, which was attributed to signal noise. We also used other correlation coefficient thresholds (i.e., 0.15, 0.2, 0.3, 0.35) for DC calculation to investigate whether the subsequent SVM results preserved (see Supplementary Materials). Then, standardized binarized DC maps were acquired by dividing by the global mean of DC values. Finally, the DC images were smoothed using a 6-mm full-width half-maximum (FWHM) isotropic Gaussian filter.

### Statistical analysis

We divided the 2-year follow-up participants into two groups: the remitted patients, who were the 9 patients with a CAPS improvement of 50% or greater, and the persistent patients who were the 11 patients with <50% improvement according to previous studies (35, 36). To determine how accurately individual patients could be classified into those two groups on the basis of preprocessed imaging datasets, a binary SVM was used as implemented in PRoNTo (http://www.mlnl.cs.ucl.ac.uk/pronto/) running under MATLAB (MathWorks, Inc., USA). We used ALFF, DC, and combined ALFF and DC information to train a linear SVM with a Gaussian kernel, respectively. Similar to other studies, we used the default parameter C = 1, which is recommended for high-dimensional data and relatively small sample sizes (15, 16). After feature space calculation, features were mean-centered, and cross-validation was performed based on a leave-one-subject-out scheme, indexed using the total accuracy and class accuracy (representing the sensitivity and specificity). The significance of both indices was estimated using a permutation test whereby the input-target data were randomly paired and the SVM rerun 1,000 times. Finally, the receiver operating characteristic (ROC) curve was plotted and the area under ROC curve (AUC) was calculated to illustrate the performance of classification.

In this study, each voxel carries a certain weight value representing its contribution toward the classification function since the input space is voxel space (one dimension per voxel). The larger the absolute magnitude of a weight vector is, the stronger it affects the final discrimination (37). Therefore, a discrimination map showing the global spatial pattern by which the groups differ could be generated. Because of the multivariate character of the SVM classifier and the discrimination is based on the whole brain pattern (all voxels contribute to the classification), local inferences should not be made from the weights. For ease of visualization, by setting the threshold to 30% of the maximum (absolute) weight value for all successful SVM-derived weight maps (16, 37), we obtained a spatial representation of the regions that contributed most to the group discrimination.

## Results

### Demographics and clinical scores

The demographic information and psychological variables in PTSD before and after treatment and at follow-up are shown in Table 1. Three months later, 21 patients responded to the pharmacotherapy with a CAPS improvement of at least 50%, and only one patient did not respond to the pharmacotherapy with a CAPS decrease by three scores. Compared to baseline, the patient group showed significant differences in CAPS, HAMD, and HAMA after treatment (paired *t*-test, *p* < 0.001) (Table 1). In the first month of the study, 12 patients reported dry mouth, eight reported constipation, five reported drowsiness, three reported dizziness, three reported hyperhidrosis, two reported palpitation, one reported headache, and one reported tinnitus, all of which gradually diminished without use of any additional drugs. At the 2-year follow-up, 9 patients were in remission, with a CAPS improvement of 50% or greater, 11 patients had persistent PTSD with less than a 50% improvement.

**Table 1:** Participant characteristics before, after treatment, and at follow-up.

**Variables (mean ± *SD*)**	**Pre-treatment**	**Post-treatment**	**Follow-up remitted**	**Follow-up persistent**	***p*-value (pre-treatment vs. post-treatment)**	***p*-value (remitted vs. persistent)**
Gender(f/m)	22 (17/5)	22 (17/5)	9 (8/1)	11 (7/4)	_	0.319^a^
Age(yrs)	45.8 ± 7.0	45.8 ± 7.0	46.2 ± 6.8	47.9 ± 7.0	_	0.593
Education (yrs)	6.8 ± 3.3	6.8 ± 3.3	6.7 ± 3.3	7.4 ± 2.8	_	0.616
CAPS	67.3 ± 14.5	16.4 ± 13.7	21.7 ± 9.4	62.6 ± 25.0	<0.001	<0.001
HAMD	19.5 ± 8.6	5.8 ± 6.6	_	_	<0.001
HAMA	17.0 ± 7.6	7.4 ± 8.8	_	_	<0.001
CGI-S	5.1 ± 0.7	1.7 ± 0.9	_	_	_
CGI-I	_	1.2 ± 0.5	_	_	_

*CAPS, Clinician Administered Posttraumatic Stress Disorder Scale; HAMD, the Hamilton Rating Scale for Depression. HAMA, the Hamilton Rating Scale for Anxiety; CGI-S, Clinical global impressions severity; CGI-I, Clinical global impressions-improvement*.

a *Fisher's Exact Test*.

### Classification performance

**Figure 1:**
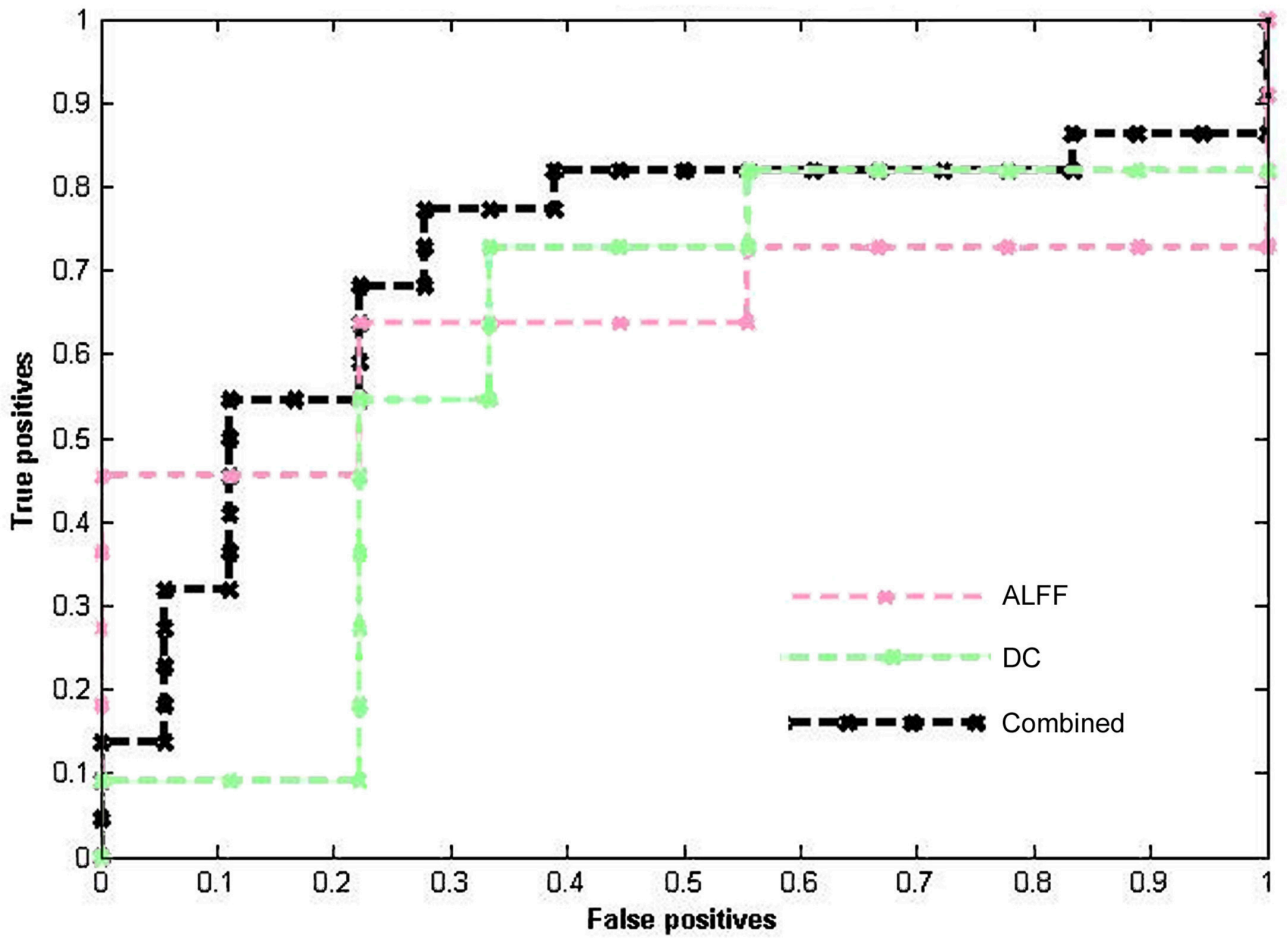
ROC curves of different methods show the trade-off between the true positives/sensitivity (y-axis) and false positives/specificity (x-axis, 1-specificity).

Table 2 lists the classification results of the single-feature method and our multi-level feature combination method (i.e., combined ALFF and DC information). The multi-level feature combination approach resulted in an accuracy of 72.50% (*p* = 0.004) at pre-treatment, with a sensitivity of 66.67%, and a specificity of 77.27%. The single-feature classification (when using ALFF or DC) failed to survive the permutation test (*p* > 0.05). The binary and linear prediction of long-term therapeutic response by combined ALFF and DC information obtained before treatment yielded accuracy rates significantly above the level of chance. However, long-term treatment outcome could not be successfully classified by post-treatment imaging features with accuracy rates <50% (*p* > 0.05). The corresponding ROC curves were plotted (see Figure 1). The larger the area under the ROC is obtained, the better the classification performance.

**Table 2:** Prediction of Long-term Clinical Outcome by Multimodal Imaging Obtained before and after Treatment.

**Modality**	**Pre-treatment**					**Post-treatment**				
	**TA (%)**	**SEN (%)**	**SPE (%)**	**AUC value**	***p*-value**	**TA (%)**	**SEN (%)**	**SPE (%)**	**AUC value**	***p*-value**
ALFF	65.00	66.67	63.64	0.64	0.074	40.00	22.22	54.55	0.25	0.677
DC	65.00	55.56	72.73	0.61	0.073	55.00	44.44	63.64	0.56	0.207
Combined	72.50	66.67	77.27	0.72	0.004	47.50	27.78	63.64	0.45	0.488

*TA, total accuracy; SEN, sensitivity; SPE, specificity; AUC, area under receiver operating characteristic curve*.

### The most discriminative regions

**Figure 2:**
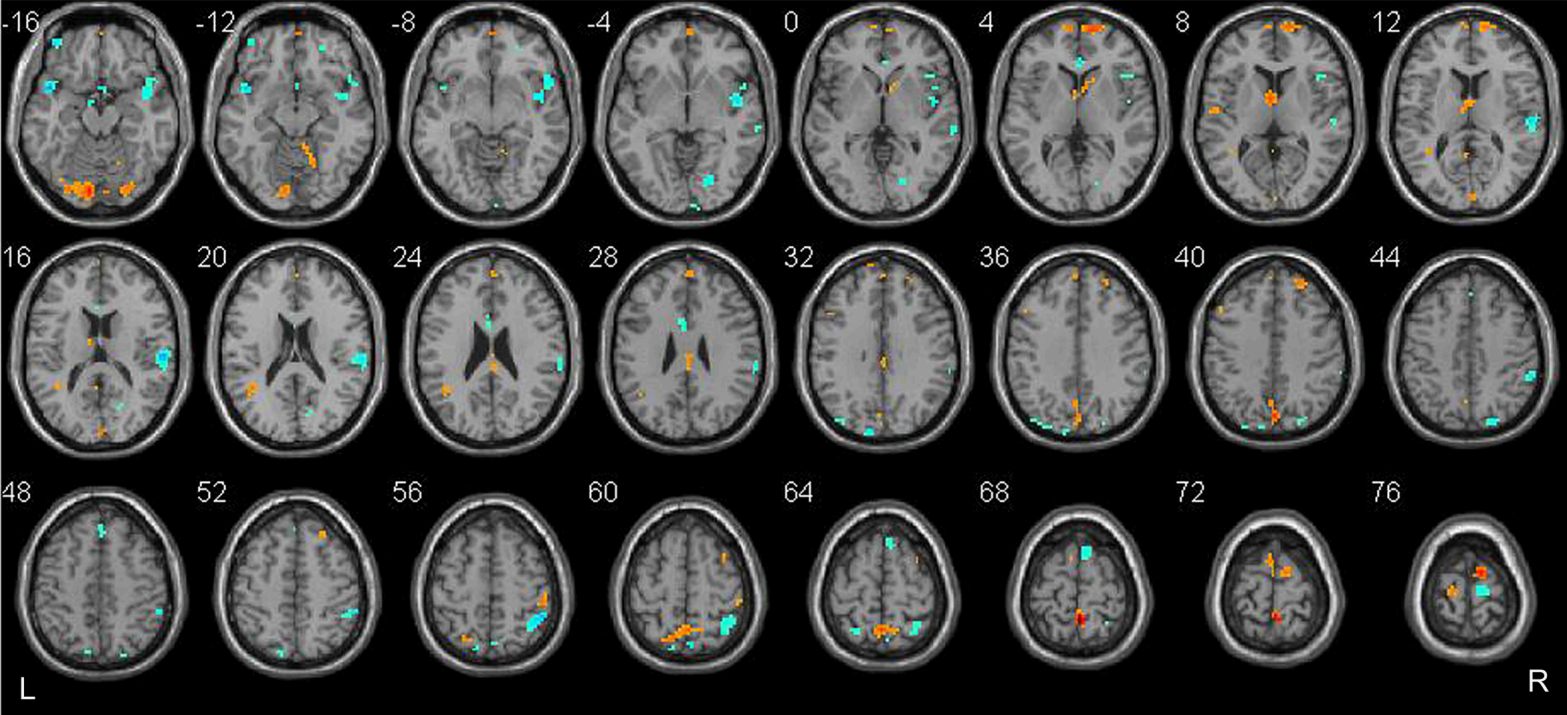
Brain regions which showed the highest prognostic value of integrated ALFF and DC features. These regions were identified by setting the threshold to the top 30% of the weight vector scores. Red indicates higher values in the remitted than the persistent patients, while blue indicates higher values for the persistent than the remitted patients.

Classification was based on functional alterations across the whole brain (Figure 2). In comparison of remitted patients and persistent patients, regions displaying most difference in combined ALFF and DC appeared in the bilateral precuneus, bilateral superior temporal area, bilateral insula, bilateral dorsal medial prefrontal cortex (dmPFC), right frontal orbital cortex, right supplementary motor area, bilateral lingual gyrus, and bilateral Cerebelum_Crus1 (see Table 3 for a full list).

**Table 3:** The most discriminating regions revealed by combined ALFF and DC discriminative map (in the Top 30% of the maximum absolute weight vector score), for the comparison between remitted patients and persistent patients.

**Region**	**Number of voxels**	**MNI coordinate (x, y, z)**	***w_*i*_* (× 10^−2^)**
**REMITTED PATIENTS > PERSISTENT PATIENTS**			
Cerebellum_Crus1_L / Lingual_L	176	−9, −87, −15	1.92
Cerebellum_Crus1_R / Lingual_R / Inferior Occipital Gyrus_R	95	27, −84, −18	1.84
Cerebellum_Crus1_R	20	9, −87, −21	1.63
dmPFC_R	61	15, 63, 3	1.72
dmPFC_L	22	0, 66, 18	1.44
Bilateral dmPFC	22	0, 54, 30	1.15
Bilateral precuneus	44	3, −78, 39	1.78
	86	3, −48, 69	3.16
Frontal_Sup_R/ Supp_Motor_Area_R	21	12, −3, 75	2.00
**REMITTED PATIENTS < PERSISTENT PATIENTS**			
Superior temporal area/ Frontal Orbital Cortex_R	28	36, 21, −21	−2.26
Superior temporal area_L / left insula	27	−45, 9, −15	−2.23
Superior temporal area_R / right insula	100	48, 0,−3	−1.71
Fusiform_R/Lingual Gyrus_R	21	21, −75, −3	−1.50
Right Superior temporal area	200	60, −21, 15	−2.06
Supp_Motor_Area_R	38	6, 24, 66	−1.48
Supp_Motor_Area_R / Precentral Gyrus_R	24	9, −18, 78	−1.71

*The w_i_ Refers to The Peak Weight Vector Score in Each Cluster.dmPFC, dorsal medial prefrontal cortex; Supp_Motor_Area, supplementary motor area; L, left; R, right*.

## Disscusion

In the present longitudinal rs-fMRI study, we investigated the potential alterations of brain function related to the long-term treatment outcome of paroxetine that was taken 2 years ago in individuals with PTSD. To the best of our knowledge, this is the first study to examine the prognostic value of rs-fMRI in relation to the long-term clinical outcome in PTSD. By applying an SVM method to the pre-treatment fMRI data, we successfully discriminated the remitted patients from the persistent patients with 72.50% accuracy (*p* < 0.005). Moreover, we demonstrated that the performance of the classification could be significantly improved using the combined ALFF and DC features. The most informative voxels for prognostic value were mainly located in the precuneus, superior temporal area, insula, dorsal medial prefrontal cortex (dmPFC), frontal orbital cortex, supplementary motor area, cerebellum and lingual gyrus, which have been consistently reported as important brain regions in the pathophysiology of PTSD.

The ability to advise psychiatrists and patients accurately regarding the chances of successful pharmacotherapy is of considerable value, particularly because pharmacotherapy is a time-consuming procedure (38) and has some adverse effects (39). The binary pattern classification analyses based on the combined features of ALFF and DC yielded accuracy rated as great as 72.50% accompanied by an AUC of 0.72 in the present study, which was efficient to provide preliminary support to develop the prognostic aid. However, SVM based on ALFF or DC alone did not reach sufficient overall predictive accuracies (65.0%) in the therapeutic outcome of PTSD patients, nor did it pass through the permutation test. The combined ALFF and DC measures represents both the functional segregation of the brain and the integration within the brain (23). These results suggested that single-level features could only afford limited information for discrimination of clinical outcome. Information from different levels of features may complement each other and potentially improve the prediction accuracy. Previous studies highlighted a pattern of brain activation that might predict response to a short-term PTSD treatment without reference to the long-term prognosis (40, 41). Therefore, our study not only confirmed that functional neuroimaging data has the potential to serve as prognostic biomarkers, but also provided further evidence that the multi-level rsfMRI method could help support a predictability of treatment outcome in the long run, which may be particularly important to guide personalized treatment decisions.

Contrary to the hypothesis, our study demonstrated the value of pre-treatment MRI for predicting clinical outcome; however, the post-treatment functional MRI was not able to predict clinical outcome (permutation test, *p* > 0.05) in the SVM. Changed brain function after short-term treatment of SSRIs has been observed frequently in a number of previous functional imaging studies of PTSD (9–11, 42). In our previous study, which used the same sample as in the present study, we have found that the abnormal ALFF of OFC and precuneus at pre-treatment was normalized compared to traumatized healthy controls at post-treatment (10). The OFC and the precuneus are among the most discriminative brain regions using pre-treatment rs-fMRI data in the present study. In other words, some of the brain activity or network information that could be used to discriminate the clinical outcome was interfered after SSRI treatment. Furthermore, taking medication alone can make a difference on the classification performance of post-treatment rs-fMRI data, as there were side effects from the medication. Studies involving both pre- and post-treatment fMRI data using different treatment methods are needed to replicate the findings.

The brain regions that showed the highest prognostic value (i.e., the most substantial contribution to the SVM decision function), by setting a threshold to the top 30% of the weight vector scores, are presented in Figure 2 and listed in Table 3. Interpretation of these results must take the multivariate nature of the SVM method into account. There are two possible reasons for an individual region to display high discriminative power: first, a difference in ALFF/DC between groups in that region and, second, a difference in the correlation between that region and other areas between groups. Results from multivariate methods such as SVM should not only be interpreted as individual regions but also as a spatially distributed pattern. The most discriminative regions we identified by SVM were widespread and not restrict to particular brain regions in the present study. Two discriminating patterns were revealed: first, the precuneus, dmPFC, lingual gyrus, the right supplementary area, and the Cerebelum_Crus1 showed a strong contribution favoring remitted over persistent patients with PTSD. Second, the superior temporal area, the insula, the right frontal orbital cortex, the right supplementary motor cortex, the right lingual gyrus, and fusiform gyrus showed a strong contribution favoring persistent patients over remitted ones.

The precuneus and dmPFC were core regions of the default mode network (DMN) (43, 44). Recently, the cerebellum_Crus1 was demonstrated to be associated with emotion processing (45–48) and cognitive function (49). Moreover, rs-fMRI studies have revealed that the cerebellum_Crus1 participated the DMN (50, 51). Therefore, our findings provided preliminary evidence that the DMN counts for the long-term clinical outcome of PTSD, which were supported by previous studies showing that the DMN connectivity was associated with PTSD symptom severity (52, 53). The supplementary area was believed to be among the network of neural regions mediating top-down control of negative affect in a recent meta-analysis (54) and change in the supplementary motor area over time in veterans with PTSD after paroxetine treatment was observed (11). The functional alteration of temporal lobe including the insula has been implicated in the treatment outcome of pharmacotherapy in PTSD in a number of studies (42). The insula is an important component in the salience network (55) and dysconnectivity in the salience network was thought to be related to low threshold for saliency and a hypervigilant state in PTSD (56). In addition, the anterior insula of the salience network is thought to mediate the disengagement of the DMN (57). Taken together, our results suggested that the long-term clinical outcome after paroxetine treatment was best predicted by alterations in widespread networks including the default mode and salience network, providing potential biomarkers in PTSD prognosis. It may be surprising that the amygdala and the anterior cingulate cortex were not found to be the most discriminate regions, given that these regions have been associated with treatment outcome in previous fMRI studies (41). However, a recent investigation using a multivariate analytical method did not detect treatment outcome associations with these regions (11). We also speculated that this might be related to the methods we used (i.e., discrimination is based on the whole brain pattern at resting-state).

The present study has a number of important limitations. First, the sample size of this study is very small, especially for machine learning analysis, which restricted us to using more restrictive cross-validation methods. Second, the small number of males in our sample (5 males out of 22 subjects at baseline) may to some extent limit the generalizability of our results. Studies in larger samples with a more balanced female to male ratio could tackle the limitations. Third, most patients who underwent the 12-week treatment showed a significant improvement in symptoms, which was not very generalizable. This may be due to Hawthorne effect because professional treatment was out of reach for the patients and they were never medicated before. Forth, this study was not a randomized trial inclusive of a placebo or waitlist control hence it is quite difficult to attribute the benefits directly to paroxetine treatment. Although the participants reported no life events during the 2-year follow-up, other potential factors might affect the prognosis of the PTSD patients, not just the treatment. Nevertheless, the current findings do suggest a predictive pattern indicative of general recovery course of PTSD even though it is difficult to determine to what degree the findings are relevant to the medication. Finally, we failed to perform regular follow-ups after the SSRI treatment; thus, we could not discover the time when the clinical symptoms of some PTSD patients reappeared after they reported a symptom relief right after treatment. Future studies might conduct regular follow-ups of symptom severity as well as fMRI to investigate the underlying mechanisms of prognosis of PTSD further.

## Conclusion

The present study revealed that combined information from ALFF and DC data before paroxetine treatment could predict the long-term clinical outcome of PTSD, suggesting that integration of regional and integrated network measurement could yield higher accuracy in PTSD prognostic identification. Moreover, widespread networks including the default mode and salience network showed the best discriminative performance between remitted and persistent PTSD. These results add to evidence that multi-level resting-state imaging could be used to develop biomarkers of improved and more personalized treatment interventions, which can potentially improve the prognosis of PTSD.

## Author contributions

WZ, CQ, HZ, SL, and QG contributed conception and design of the study. YM, ZR, CY, YL and, MG collected and the data for the work and organized the database. MY and HZ performed the data preprocessing and statistical analysis. MY and CQ drafted the work and revised it critically. All authors contributed to manuscript revision, read and approved the submitted version.

### Conflict of interest statement

The authors declare that the research was conducted in the absence of any commercial or financial relationships that could be construed as a potential conflict of interest.
