# Supplementary material for: Pre-treatment Resting-State Functional MR Imaging Predicts the Long-Term Clinical Outcome After Short-Term Paroxtine Treatment in Post-traumatic Stress Disorder
Source: Front Psychiatry. 2018 Oct 30;9:532. doi: 10.3389/fpsyt.2018.00532 (PMC6218594; doi:10.3389/fpsyt.2018.00532)
Supplement: Supplementary file 1 [file Table_1.DOCX]

Supplementary Material

We used a single correlation coefficient threshold of 0.25 to eliminate weak correlations possibly arising from noise signal during the degree centrality (DC) calculation. To explore whether the classification results depended on the choices of correlation thresholds, we recomputed the DC maps using other five different correlation thresholds (i.e., 0.15, 0.20, 0.25, 0.30 and 0.35) and then reperformed the supportive vector machine (SVM) analysis, respectively. First, we performed SVM based on DC maps of different correlation thresholds alone respectively, and got very low predictive accuracies (<50%) for each threshold, which is in line with our original finding that single-level features could only afford limited information for discrimination of clinical outcome. Second, we performed SVM based on combined DC maps of different thresholds and ALFF maps, respectively. The classification results of combined DC and ALFF features at different threshold were presented in Table S1.

Long-term treatment outcome could not be successfully classified by combined ALFF and DC information when the correlation thresholds of DC were either smaller than 0.25 or greater than 0.25 (p > 0.05). We speculated that a low threshold was insufficient to eliminate the voxels with weak correlations attributable to signal noise and thus the classification performance of SVM based was interfered. However, high correlation threshold of DC removed neural correlation features that potentially counted for classification of the long-term treatment outcome. Since the sample size of the present study is small and statistical power is lacked, more studies using DC of different correlation thresholds as classification features are needed to do further validation.

Table S1 Prediction of long-term treatment outcome using feature combination approach of DC at different thresholds and ALFF.

| DC Threshold | Pre-treatment MRI | | | | |
| --- | --- | --- | --- | --- | --- |
|  | TA (%) | SEN (%) | SPE (%) | AUC value | p-value |
| 0.15 | 60.00 | 50.00 | 68.18 | 0.56 | 0.074 |
| 0.20 | 60.00 | 50.00 | 68.18 | 0.56 | 0.065 |
| 0.25 | 72.50 | 66.67 | 77.27 | 0.72 | 0.004 |
| 0.30 | 57.50 | 44.44 | 68.18 | 0.55 | 0.109 |
| 0.35 | 55.00 | 38.89 | 68.18 | 0.55 | 0.156 |

DC, degree centrality; ALFF TA, total accuracy; SEN, sensitivity; SPE, specificity; AUC, area under receiver operating characteristic curve.
